# Supplementary material for: Hepatitis Delta Virus histone mimicry drives the recruitment of chromatin remodelers for viral RNA replication
Source: Nat Commun. 2020 Jan 21;11:419. doi: 10.1038/s41467-020-14299-9 (PMC6972770; doi:10.1038/s41467-020-14299-9)
Supplement: Supplementary file 1 — Supplementary Information [file 41467_2020_14299_MOESM1_ESM.pdf]

Hepatitis Delta Virus histone mimicry drives the recruitment of chromatin remodelers for viral RNA replication

Natali Abeywickrama-Samarakoon et al.

Supplementary information

- n. 5 Supplementary Figures

- n. 1 Supplementary Table

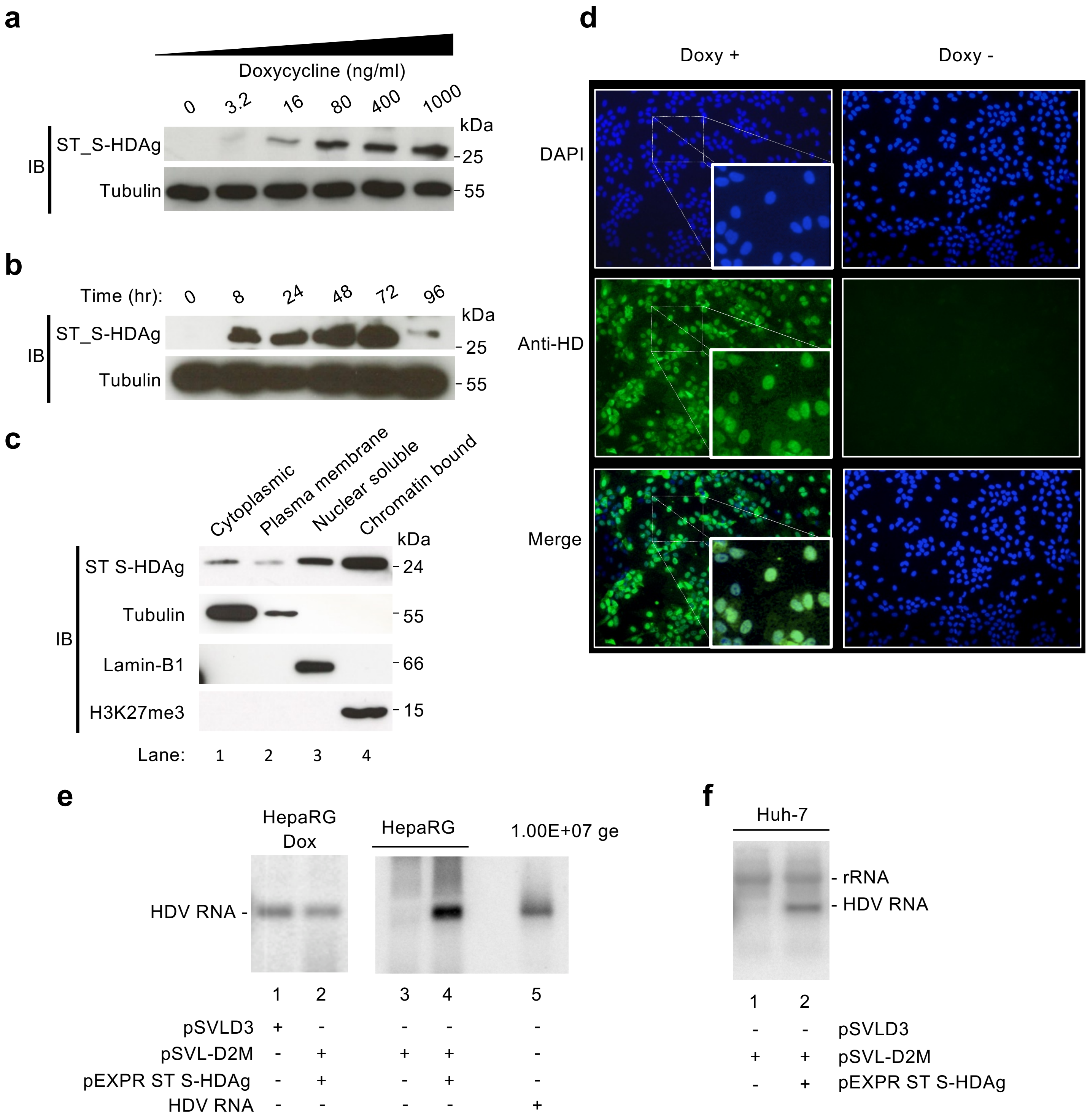

Supplementary Figure 1. Characterization of the HepaRG cell line expressing StrepTag (ST) S-HDAg protein under the control of a tetracycline inducible promoter. (**a**) Dose-dependent induction of the ST_S-HDAg protein. dHepaRG cells expressing a tetracycline inducible ST_S-HDAg protein were non-induced or induced with 3.2, 16, 80, 400, 1000 ng/ml doxycycline for 48 hours. Total protein extracts were immunoblotted and analyzed with α-Strep-Tag antibody. α-Tubulin is used as loading control. (**b**) Time-dependent induction of ST_S-HDAg protein expression. Differentiated HepaRG cells expressing the tetracycline inducible ST_S-HDAg protein were induced with 80 ng/ml doxycycline for 0, 8, 24, 48, 72, 96 hours. Total protein extracts were immunoblotted and analyzed with α-Strep-Tag antibody. α-Tubulin is used as loading control. (**c**) The ST_S-HDAg protein is predominantly localized in the chromatin bound fraction. Differentiated HepaRG cells expressing a tetracycline inducible ST_S-HDAg protein were induced with 80 ng/ml doxycycline. At 48 hours post-induction, cytoplasmic, plasma membrane, nuclear soluble and chromatin bound fractions were subjected to immunoblotting with an α-Strep-Tag antibody. Antibodies against alpha-Tubulin, Lamin-B1 and Histone H3 K27me3 were used to assess fraction purity in cytoplasmic, nuclear soluble and chromatin bound proteins, respectively. (**d**) The ST_S-HDAg protein accumulates in the nucleus. Differentiated HepaRG cells expressing a tetracycline inducible ST_S-HDAg protein were induced with 80 ng/ml doxycycline for 48 hours. Cells were subjected to indirect immunofluorescence and the ST_S-HDAg protein was detected using a rabbit α-HDAg polyclonal antibody (green). Cell nuclei were stained using DAPI (blue). (**e**) The ST-S-HDAg protein is functional in HDV replication. *Left panel:* Doxycycline-induced HepaRG cells expressing the ST-S-HDAg protein were transfected either with the replication competent pSVLD3 plasmid (lane 1) or the pSVL-D2M plasmid (lane 2). *Right panel:* HepaRG cells were transiently co-transfected with pSVL-D2M and pEXPR-ST-S-HDAg (lane 4) or empty pEXPR vector (lane 3). Lane 5: 1,00E+07 copies of genomic HDV RNA. (**f**) Huh7 cells were transiently co-transfected with pSVL-D2M and pEXPR-ST-S-HDAg (lane 2) or empty pEXPR vector (lane 1). In both (**e**) and (**f**) total RNAs were extracted 9 days post transfection and analyzed by northern blot assay using a 32P-radiolabeled probe for detection of genomic HDV RNA.

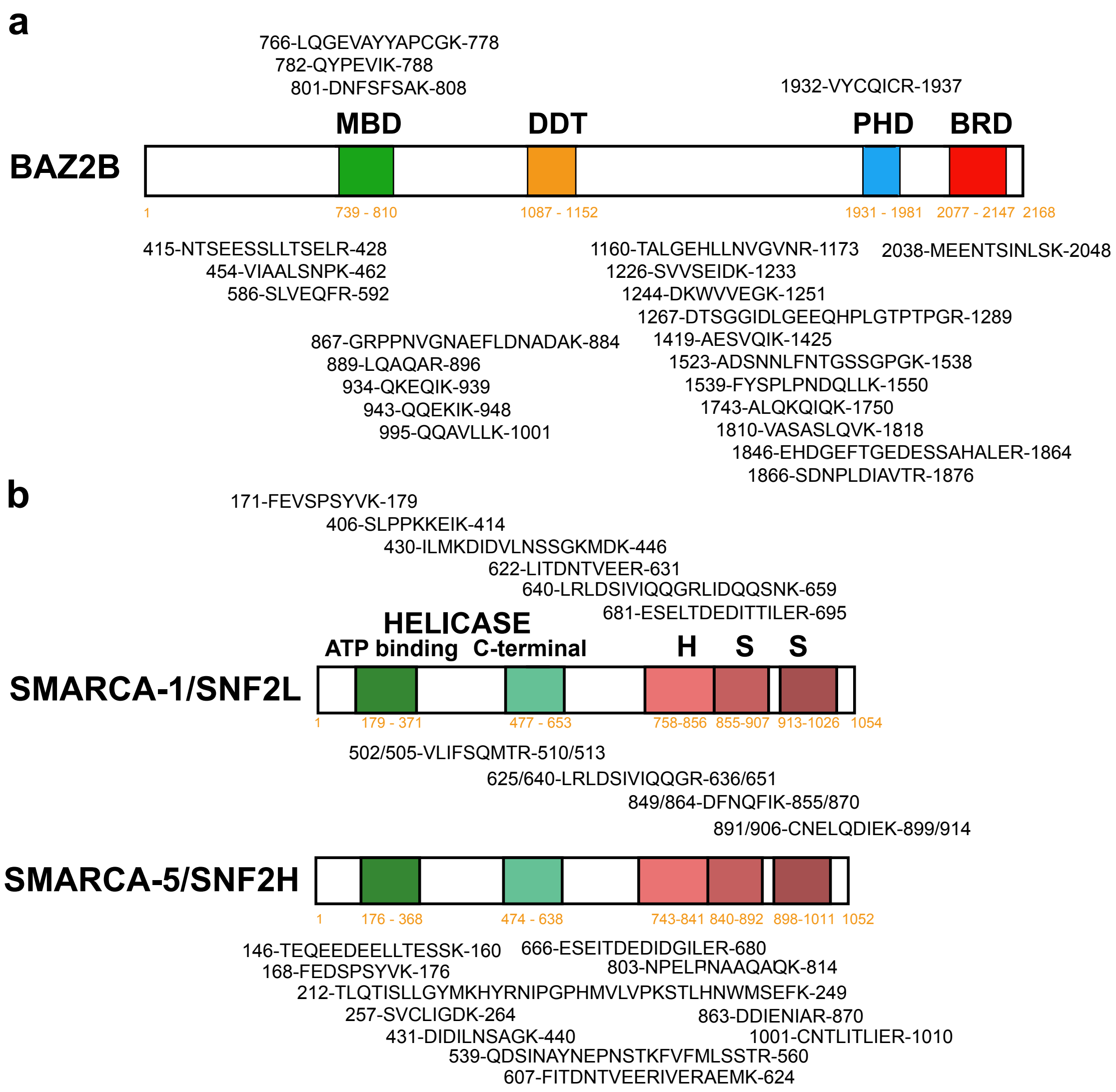

Supplementary Figure 2. Mapping of LC-MS/MS derived peptides on BAZ2B and SNF2L/H subunits of BRF chromatin remodelers. Sequence coverage of BAZ2B (Q9UIF8) (**a**) and SNF2L (P28370) or SNF2H (060264) (**b**) derived tryptic peptides identified by LC-MS/MS analysis as HDAg binding partners in differentiated HepaRg cells. In (**b**) the SNF2L and SNF2H specific peptides are displayed at the top and the bottom, respectively; the peptides displayed in the middle are common to both SNF2L and SNF2H. TAM = {Tip5/ARBP/MBD} domain, DDT = DNA Biding homeobox and Different Transcription factors, PHD = Plant Homeo Domain, BRD = Bromo Domain, HSS = HAND/SAND/SLIDE domains.

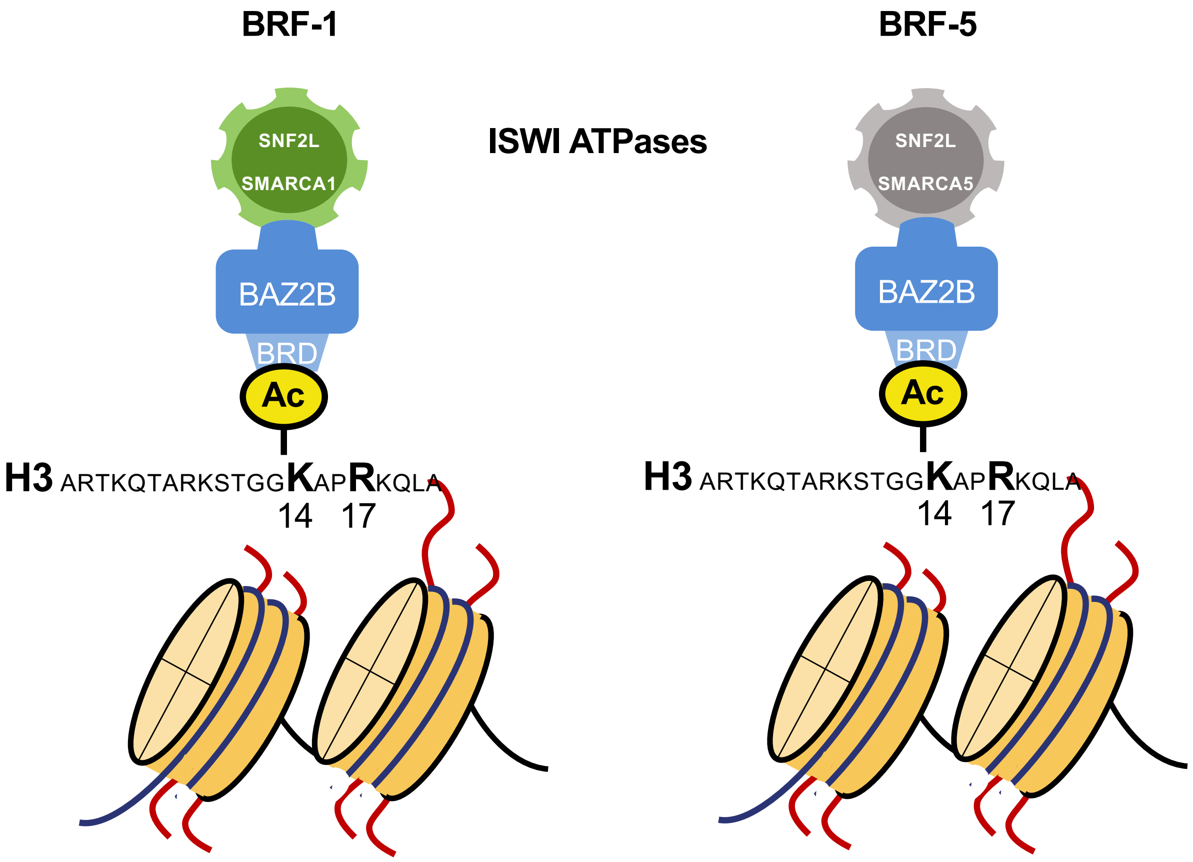

Supplementary Figure 3. Schematic representation of BRF chromatin remodelers. BRF complexes comprise the accessory subunit BAZ2B and the ISWI ATPases SNF2L/SMARCA1 (BRF-1 complex) or SNF2H/SMARCA5 (BRF-5 complex). The BAZ2B bromodomaine (BRD) specifically recognizes histone H3 K14ac. ISWI = Imitation SWItch.

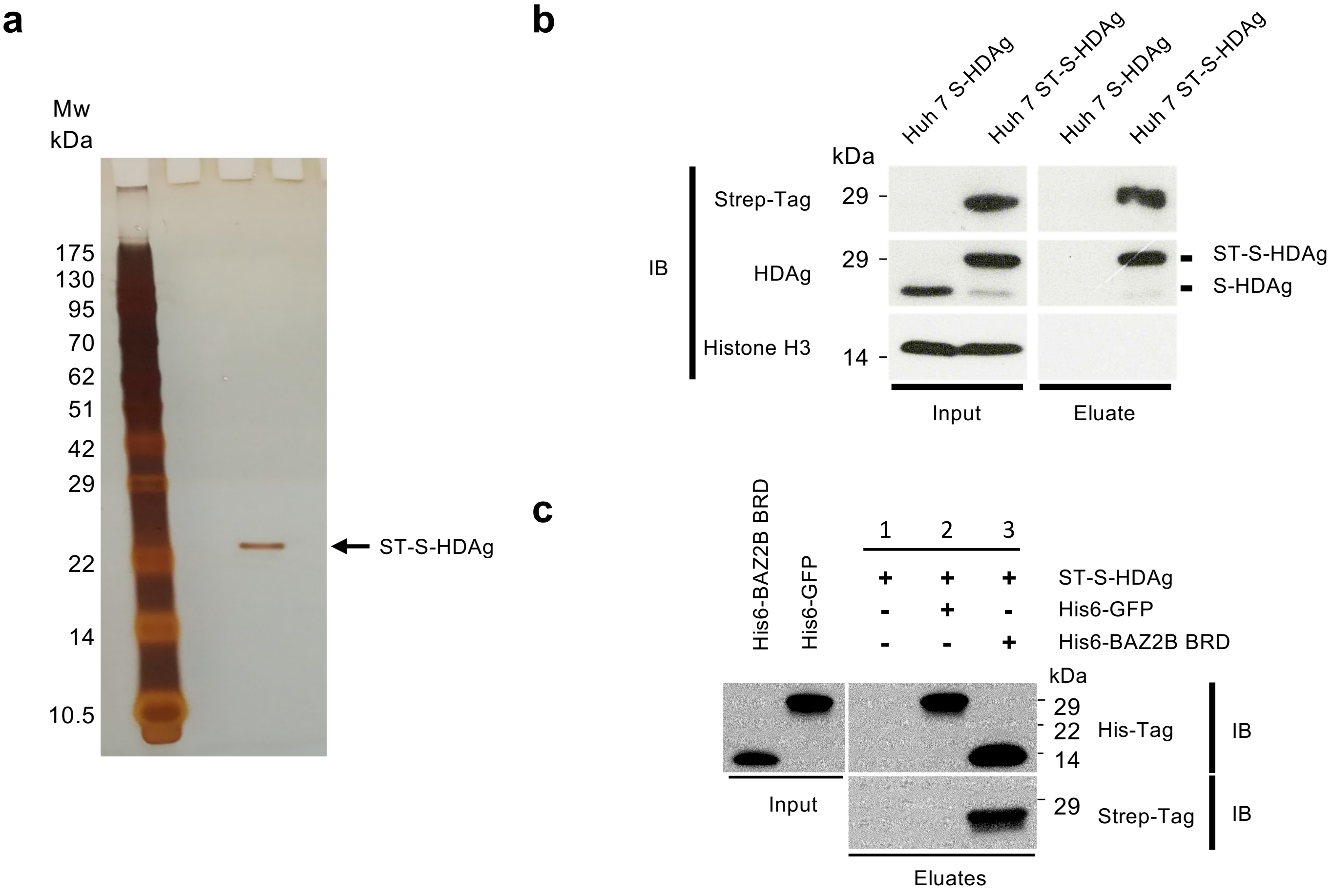

Supplementary Figure 4. ST-S-HDAg affinity purification on Strep-Tactin® coated beads. (**a**) A nuclear extract prepared from Huh 7 cells expressing ST-S-HDAg was subjected to affinity chromatography on a Strep-Tactin®-XT resin. The purity of ST-S-HDAg was verified by silver staining after SDS-PAGE separation. Mw, molecular mass markers. (**b**) ST-S-HDAg binds specifically to the StrepTactin®-XT beads. Huh7 cell nuclear extracts expressing either S-HDAg or ST-S-HDAg were subjected to affinity chromatography on a Strep-Tactin®-XT resin. Five percent of the input and 10% of the SDS-eluates were subjected to western blotting and analyzed with antibodies directed against Strep-Tag, HDAg or Histone H3. (**c**) Pull-down assay of His-Tag BAZ2B BRD and ST-S-HDAg. Ni-NTA beads bound His-Tag BAZ2B BRD (0.5 µM) or His-Tag GFP (0.5 µM) were mixed with purified ST-S-HDAg. 80% of the SDS-eluate was subjected to immunoblotting with the anti-Strep-Tag antibody whereas 3% of the SDS-eluate was subjected to immunoblotting with anti His-Tag antibody.

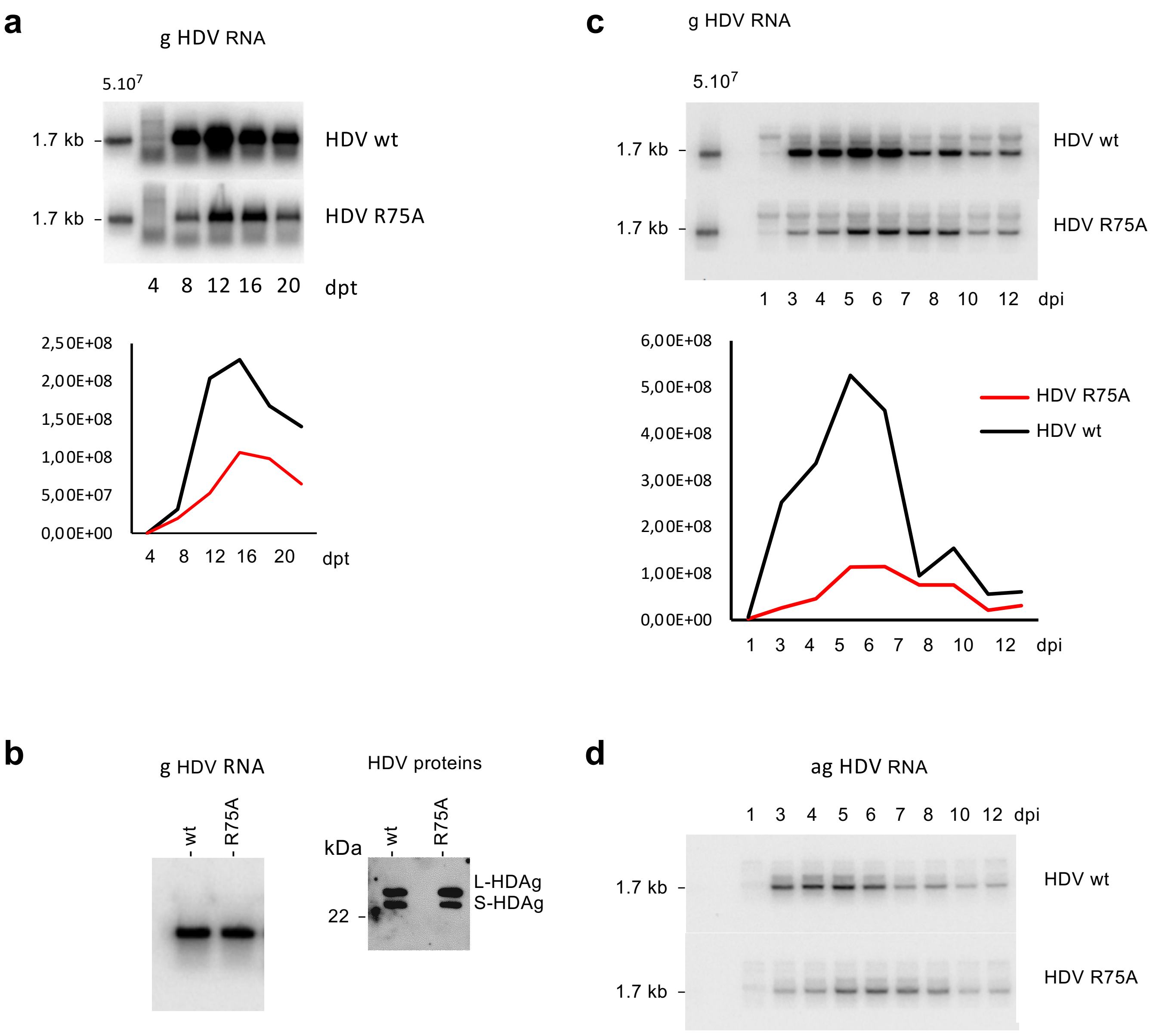

Supplementary Figure 5. Recombinant virus production, calibration and infection of the Huh7-106 cell line. (**a**) Viral production. Huh7 cells were transfected with pCDNA3-HDV1.3wt or pCDNA3-HDV1.3R75A and pT7HB2.7. Supernatant was collected every 4 day for 20 days. Total RNA was submitted to northern blotting using an antigenomic ^{32}P-riboprobe to detect viral genome. (**b**) Viral preparation was calibrated to 10^9 genome equivalent per ml. Northern blot and and immunblot were performed on viral preparations to standardize the inoculum for both viruses. (**c**) Viral infection of Huh7-106 cell line. Both wt and R75A HDV particles were inoculated to the Huh7-106 cell line. Total cell RNA was extracted at the indicated times and analyzed by northern blotting. The curves represent the kinetic of the experiment comparing the wt and R75A g HDV RNAs in the cells, indicating that at day 5 the level of R75A virus was 5 times less than the wt. (**d**) Antigenomic (ag) HDV RNA was detected using a genomic riboprobe in total cell RNA prepared as in (**c**).

Supplementary Table 1

Primers List

Oligonucleotides	References	Identifier
HDV Forward<br>5'-TGGACGTGCGTCCTCCT-3'	1	N/A
HDV Reverse<br>5'-TCTTCGGGTCGGCATGG-3'	1	N/A
HDV-835-851<br>5'-TGGACGTGCGTCCTCCT-3'	2	N/A
HDV-905-889<br>5'-TCTTCGGGTCGGCATGG-3'	2	N/A
Biotinylated HDV Forward<br>5'-biotin GCGCCGGCYGGGCAAC-3'	2	N/A
Biotinylated HDV Reverse<br>5'- biotin-TTCCTCTTCGGGTCGGCATG-3'	2	N/A
SNF2L Set A Forward<br>5'- TTTGGAAGATTATTGCATGTGGC-3'	3	N/A
SNF2L Set A Reverse<br>5'- TTGTAGATCAACCTGTGGGTTCC -3'	3	N/A
SNF2L Set B Forward<br>5'- CTTCTGGCAAGATGGACAAGATG -3'	3	N/A
SNF2L Set B Reverse<br>5'- CTTTGACCCAGAAATTCCACTTC -3'	3	N/A
GAPDH Forward<br>5' ATGAGAAGTATGACAACAGCCTCAAGAT-3'	4	N/A
GAPDH Reverse<br>5'-ATGAGTCCTTCCACGATACCAAAGTT-3'	4	N/A
RPLP0 Forward<br>5'-AGCCCAGAACACTGGTCTC-3'	5	N/A
RPLP0 Reverse<br>5'-ACTCAGGATTTCAATGGTGCC-3'	5	N/A

Supplementary References

1. Beilstein, F., Blanchet, M., Vaillant, A. & Sureau, C. Nucleic Acid Polymers Are Active against Hepatitis Delta Virus Infection In Vitro. *J. Virol.* **92**, (2018).
2. Giersch, K. *et al.* Both interferon alpha and lambda can reduce all intrahepatic HDV infection markers in HBV/HDV infected humanized mice. *Sci. Rep.* **7**, 3757 (2017).
3. Barak, O., Lazzaro, M. A., Cooch, N. S., Picketts, D. J. & Shiekhattar, R. A tissue-specific, naturally occurring human SNF2L variant inactivates chromatin remodeling. *J. Biol. Chem.* **279**, 45130–45138 (2004).
4. Chujo, T. *et al.* Unusual semi-extractability as a hallmark of nuclear body-associated architectural noncoding RNAs. *EMBO J.* **36**, 1447–1462 (2017).
5. Li, R., Harvey, A. R., Hodgetts, S. I. & Fox, A. H. Functional dissection of NEAT1 using genome editing reveals substantial localization of the NEAT1_1 isoform outside paraspeckles. *RNA N. Y. N* **23**, 872–881 (2017).
