# Supplementary material for: Hepatitis Delta Virus histone mimicry drives the recruitment of chromatin remodelers for viral RNA replication
Source: Nat Commun. 2020 Jan 21;11:419. doi: 10.1038/s41467-020-14299-9 (PMC6972770; doi:10.1038/s41467-020-14299-9)

Figure 1. The chromatin remodeling complexes BRF1 and BRF5 interact with the HDV RNP

Fig. 1a Interaction of wt SHDAg with endogenous SNF2L, SNF2H and BAZ2B.

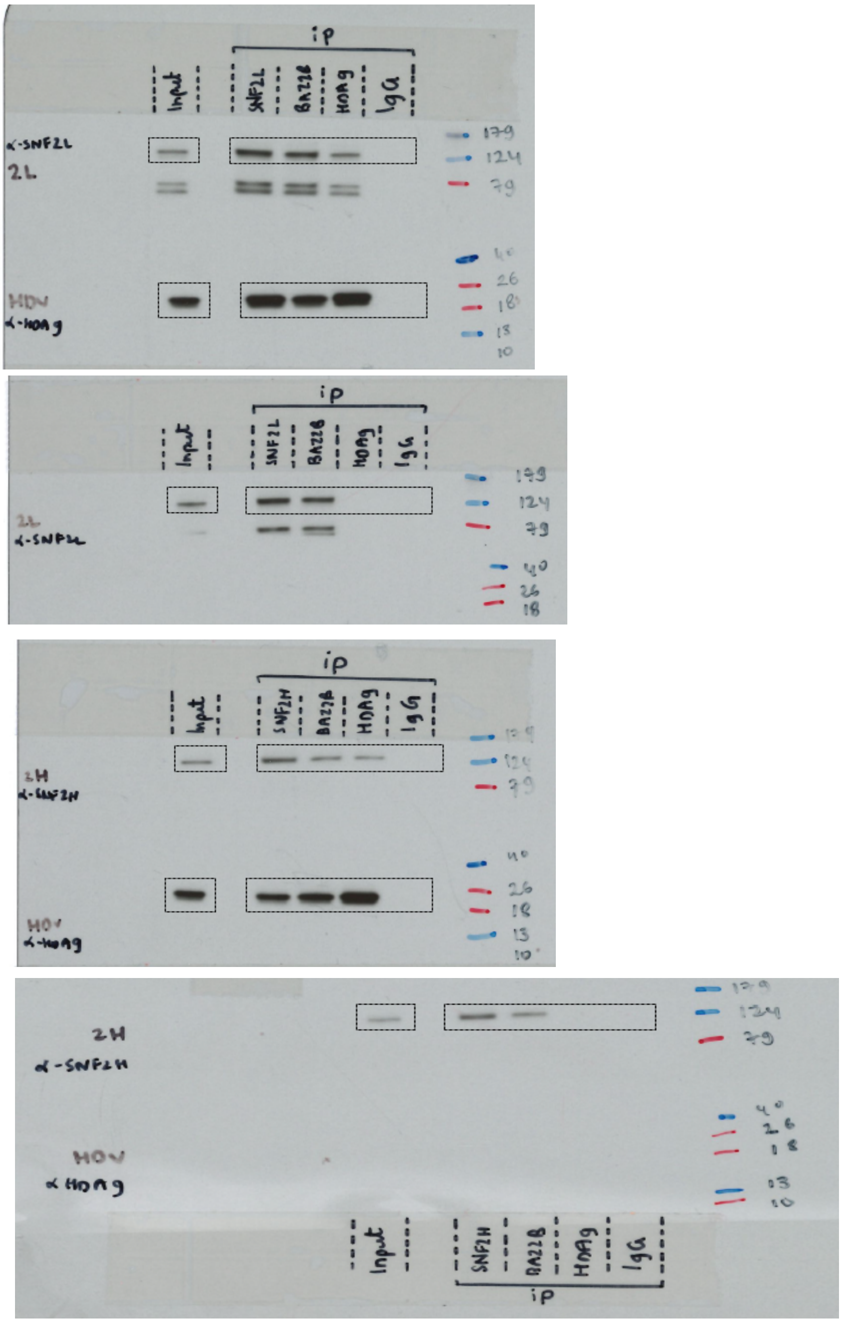

Figure 1. The chromatin remodeling complexes BRF1 and BRF5 interact with the HDV RNP

Fig. 1b Amplification of SNF2L variants from hepatocyte derived cell lines.

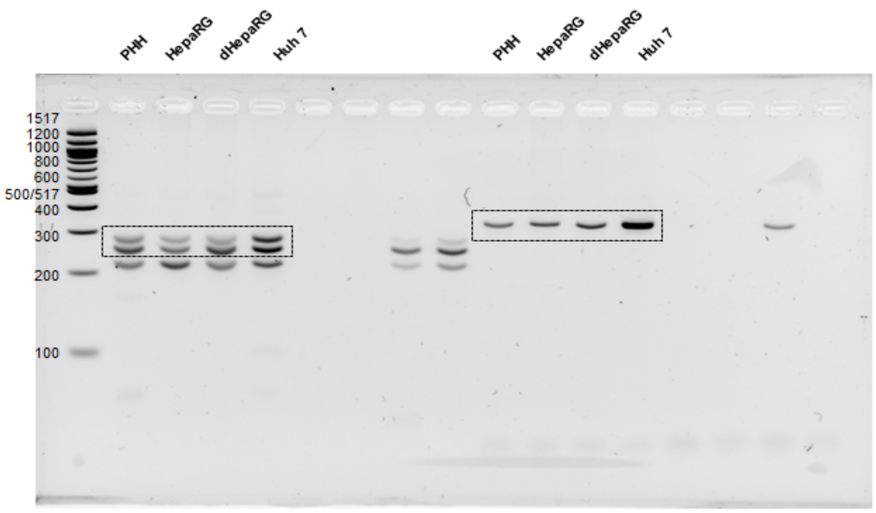

Figure 1. The chromatin remodeling complexes BRF1 and BRF5 interact with the HDV RNP

Fig. 1c HDV RNA immunoprecipitation assays (Huh 7)

	% input				
	Dataset 1	Dataset 2	Dataset 3	Mean	SEM
HDAg	66,4	51,3	70,25	62,65	5,78280497
Pol II	17,4	17,8	17	17,4	0,23094011
SNF2L	17,9	9,9	17,903	15,2343333	2,66716681
SNF2H	4,2	2,5	2,09	2,93	0,64593601
BAZ2B	7,8	8	4,88	6,89333333	1,00832094
H3	0,3	0,3	0,3	0,3	0

Figure 1. The chromatin remodeling complexes BRF1 and BRF5 interact with the HDV RNP

Fig. 1d HDV RNA immunoprecipitation assays (PHH)

	% input				
	Dataset 1	Dataset 2	Dataset 3	Mean	SEM
HDAg	41,7	38,5	38,4	39,5333333	1,08371788
Pol II	8,6	8,5	9,87	8,99	0,44094595
SNF2L	16,6	12,4	32,8	20,6	6,21932472
SNF2H	7,9	6,7	16,8	10,4666667	3,18555769
BAZ2B	8,5	9,14	14	10,5466667	1,73652271
H3	0	0	0	0	0

Figure 1. The chromatin remodeling complexes BRF1 and BRF5 interact with the HDV RNP

Fig. 1e Genomic HDV RIP assay

	% Input					
	Dataset 1	Dataset 2	Dataset 3	Dataset 4	Mean	SEM
HDAg	6,93393964	13,6944841	18,2415342	37,1852383	19,0137991	6,48726773
Pol II	3,21020242	4,13601359			3,673108	0,46290559
SNF2L	0,4834148	0,99918588	2,37655899	1,02705889	1,22155464	0,40477998
SNF2H	0,82221039	1,11080683	0,44137169	0,65960048	0,75849734	0,14098776
BAZ2B	0,05733281	0,32804742	1,17118793	3,03695919	1,14838184	0,67271719
H3	0	0	0	0	0	0

Figure 1. The chromatin remodeling complexes BRF1 and BRF5 interact with the HDV RNP

Fig. 1f Antigenomic HDV RIP assay

	% Input					
	Dataset 1	Dataset 2	Dataset 3	Dataset 4	Mean	SEM
HDAg	10,7933851	9,85300516	2,9482294	6,51237659	7,52674906	1,78124891
Pol II	14,1397606	3,16034039			8,65005051	5,48971011
SNF2L	2,56593339	1,1039913	0,93908429	0,41316409	1,25554327	0,46096269
SNF2H	0,36101827	0,36836793	0,30141778	0,30141778	0,33305544	0,01832752
BAZ2B	0,49641817	0,39131449	0,47094282	0,42134856	0,44500601	0,0237318
H3	0	0	0	0	0	0

Figure 2. BAZ2B enhances HDV replication and its BRD interacts with S-HDAg.

Fig. 2a Decrease in HDV RNA replication in PHHs transduced with shRNAs against BAZ2B

	Relative BAZ2B mRNA expression					
	Dataset 1	Dataset 2	Dataset 3	Dataset 4	Mean	SEM
Scramble shRNA	1,010549536	1,01113921	1,00447095	1,01999936	1,012	0,003197
BAZ2B shRNA #1	0,4061262	0,36014922	0,66741993	0,82169031	0,5638	0,1094
BAZ2B shRNA #2	0,77736641	0,3959344	0,59186224	0,79553648	0,6402	0,09352
BAZ2B shRNA #3	0,76136844	0,65671228	0,54714685	0,45691573	0,6055	0,06608

Dunn's multiple comparisons test	Mean rank diff.	Significant?	Summary	Adjusted P Value
Scramble shRNA vs. BAZ2B shRNA #1	8,5	Yes	*	0,0347
Scramble shRNA vs. BAZ2B shRNA #2	7,25	No	ns	0,0938
Scramble shRNA vs. BAZ2B shRNA #3	8,25	Yes	*	0,0428

Figure 2. BAZ2B enhances HDV replication and its BRD interacts with S-HDAg.

Fig. 2b Decrease in HDV RNA replication in PHHs transduced with shRNAs against

	Relative HDV RNA expression					
	Dataset 1	Dataset 2	Dataset 3	Dataset 4	Mean	SEM
Scramble shRNA	1,000678007	1,00230515	1,00043129	1,00130385	1,001	0,0004671
BAZ2B shRNA #1	0,150456256	0,5015972	0,16229232	0,61985385	0,3585	0,1192
BAZ2B shRNA #2	0,234583984	0,29244033	0,25115792	0,50697974	0,3213	0,06308
BAZ2B shRNA #3	0,592711787	0,20075601	0,76843759	0,39410902	0,489	0,1228

Dunn's multiple comparisons test	Mean rank diff.	Significant?	Summary	Adjusted. P Value
Scramble shRNA vs. BAZ2B shRNA #1	9	Yes	*	0,0225
Scramble shRNA vs. BAZ2B shRNA #2	8,5	Yes	*	0,0347
Scramble shRNA vs. BAZ2B shRNA #3	6,5	No	ns	0,1605

Figure 2. BAZ2B enhances HDV replication and its BRD interacts with S-HDAg.

Fig. 2c PHHs survival was assessed by performing the Red Neutral assay following transduction with BAZ2B shRNAs

	Cell viability (% of control)				
	Non-infected				
	Dataset 1	Dataset 2	Dataset 3	Mean	SEM
Scramble shRN	87,12765957	87,6400299	86,74189	87,1698598	0,26012783
BAZ2B shRNA #	87,5912409	87,6737381	87,5867105	87,6172298	0,02828438
BAZ2B shRNA #	87,6589379	87,5748503	87,6029963	87,6122615	0,0247121
BAZ2B shRNA #	90,3004945	90,4	90,4271548	90,3758831	0,03850076

	Cell viability (% of control)				
	HDV infected				
	Dataset 1	Dataset 2	Dataset 3	Mean	SEM
Scramble shRN	89,92932862	86,6714697	89,4625922	88,6877969	1,017127
BAZ2B shRNA #	88,4335504	89,0909091	88,2577202	88,5940599	0,25355697
BAZ2B shRNA #	86,4321608	86,4864865	86,0623432	86,3269968	0,13325287
BAZ2B shRNA #	90,1481481	90,6959434	90,5970149	90,4803688	0,16854738

Figure 2. BAZ2B enhances HDV replication and its BRD interacts with S-HDAg.

Fig. 2d Decrease in HDV RNA replication in PHHs treated with the the BAZ2B inhibitor

	Relative HDV RNA expression								
	Dataset 1	Dataset 2	Dataset 3	Dataset 4	Dataset 5	Dataset 6	Dataset 7	Mean	SEM
DMSO	1,00229909	1,00139677	1,00347391	1,0004325	1,0005904	1,00362177	1,00046093	1,002	0,000527
GSK2801	0,59186224	0,42829201	0,3842188	0,3142533	0,39216991	0,33892888	0,23947184	0,3842	0,0417
GSK8573	0,82169031	1,21981864	1,035265	1,235419	1,15647134	1,36755906	1,39187272	1,175	0,07472

Dunn's multiple comparisons test	Mean rank diff	Significant?	Summary	Adjusted P Value
DMSO vs. GSK2801	8	Yes	*	0,0317
DMSO vs. GSK8573	-5	No	ns	0,2633

Figure 2. BAZ2B enhances HDV replication and its BRD interacts with S-HDAg.

Fig. 2e PHHs survival was assessed by performing the Red Neutral assay following treatment with either GSK2801 or GSK8573

	GSK 2801				
Concentration (µM)	Dataset 1	Dataset 2	Dataset 3	Mean	SEM
1	92,369911	96,4822695	100,787898	96,5466928	2,43027698
5	118,137622	104,756469	102,831595	108,575229	4,81337761
10	108,256543	86,3806605	96,6491724	97,0954586	6,31896451
15	81,1570782	110,21571	102,232797	97,8685282	8,66768229
20	114,163823	99,5939911	94,5724907	102,776768	5,87516321
25	89,8573693	93,6067019	91,1420205	91,5353639	1,10006258
30	131,452404	88,3548261	84,9646446	101,590625	14,9629289
40	89,2845146	117,321504	92,2073215	99,6044467	8,89861955

	GSK 8573				
Concentration (µM)	Dataset 1	Dataset 2	Dataset 3	Mean	SEM
1	100,970612	94,893617	100,346675	98,736968	1,93009797
5	113,462977	111,643836	98,9567809	108,021198	4,56253077
10	67,0475488	78,9581205	94,0250303	80,0102332	7,80547528
15	55,8823529	97,0695971	86,0175695	79,6565065	12,3077836
20	68,5153584	78,6845311	77,2118959	74,8039285	3,17289293
25	68,2646593	85,0529101	64,7144949	72,6773548	6,27207287
30	93,8174681	54,0445487	71,2690733	73,0436967	11,5156885
40	17,7695667	28,7705957	36,3537514	27,6313046	5,39495037

Figure 2. BAZ2B enhances HDV replication and its BRD interacts with S-HDAg.

Fig. 2f HDV RIP in HDV infected PHHs treated with BAZ2B inhibitor GSK2801

	DMSO (% Input)				
	Dataset 1	Dataset 2	Dataset 3	Mean	SEM
HDAg	32,580135	27,6474876	26,8115901	29,0130709	1,79978155
Pol II	23,2629929	20,2308746	19,5278987	21,0072554	1,14597957
BAZ2B	5,68337664	5,04257722	4,13151518	4,95248968	0,45024265
SNF2L	28,2083027	24,3848131	23,6791921	25,4241026	1,40692368
H3	0,2	0,2	0,2	0,2	1,9626E-17

	GSK2801 (% Input)				
	Dataset 1	Dataset 2	Dataset 3	Mean	SEM
HDAg	21,5985295	8,15111594	8,45794497	12,7358635	4,43221814
Pol II	8,09710022	3,83965457	3,49897587	5,14524355	1,47920123
BAZ2B	0	0,14515301	0,06707057	0,07074119	0,04194224
SNF2L	9,53065231	4,5539314	4,38785414	6,15747928	1,68726777
H3	0,2	0,2	0,2	0,2	1,9626E-17

Figure 2. BAZ2B enhances HDV replication and its BRD interacts with S-HDAg.

Fig. 2g Pull-down assay of Strep-Tagged S-HDAg and BAZ2B BRD

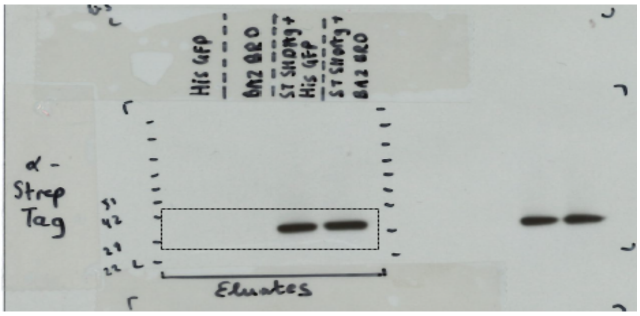

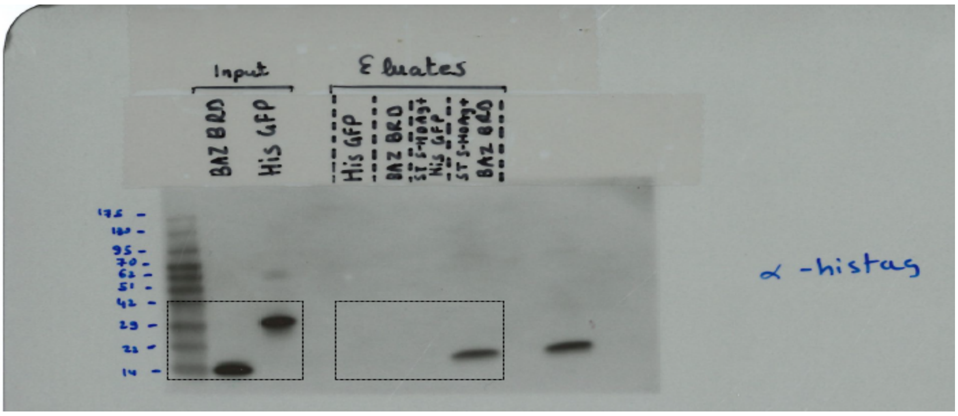

Figure 3. R75A S-HDAg mutation affects the binding to BAZ2B BRD- without altering S-HDAg localization and acetylation.

Fig. 3b Wt S-HDAg and R75A S-HDAg proteins have a similar nuclear localization pattern

Provided upon request

(The files could not be enclosed since they were too heavy to be uploaded)

Figure 3. R75A S-HDAg mutation affects the binding to BAZ2B BRD- without altering S-HDAg localization and acetylation.

Fig. 3c Wt and R75A S-HDAg are expressed at the same level

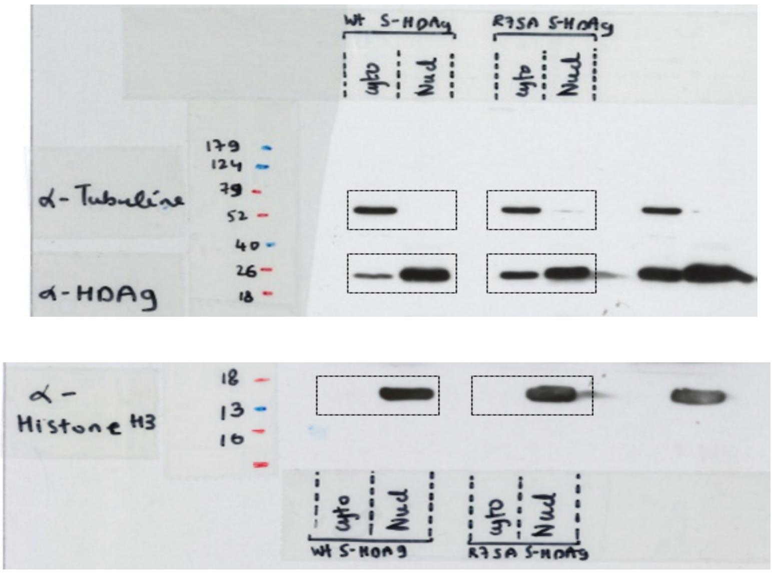

Figure 3. R75A S-HDAg mutation affects the binding to BAZ2B BRD- without altering S-HDAg localization and acetylation.

Fig. 3d Wt and R75A S-HDAg have similar acetylation levels in Huh7 cells stably expressing each proteins

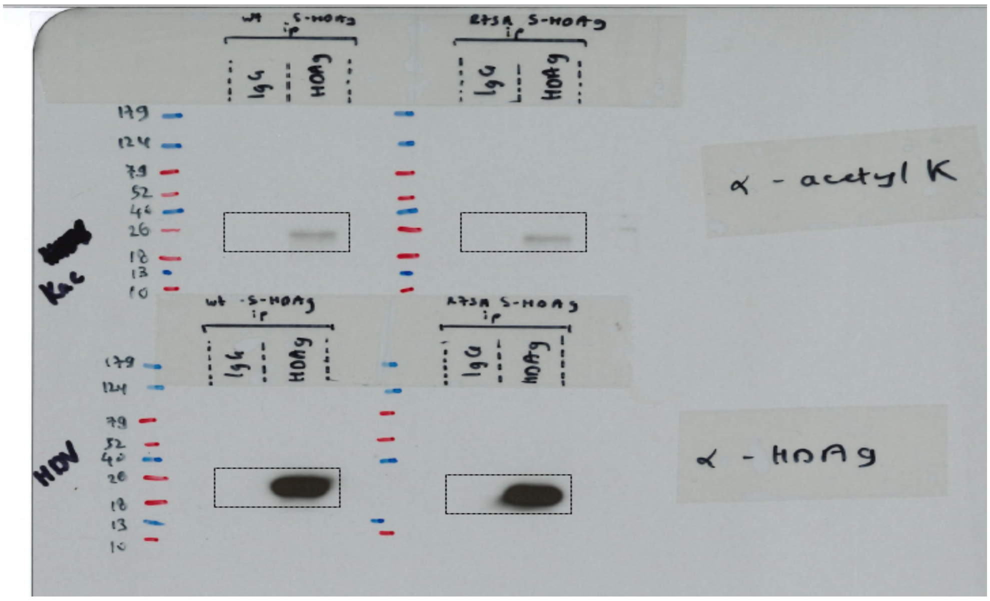

Figure 3. R75A S-HDAg mutation affects the binding to BAZ2B BRD without altering S-HDAg localization and acetylation.

Fig. 3e S-HDAg and R75A S-HDAg protein stability (Cycloheximide 100 µg/ml)

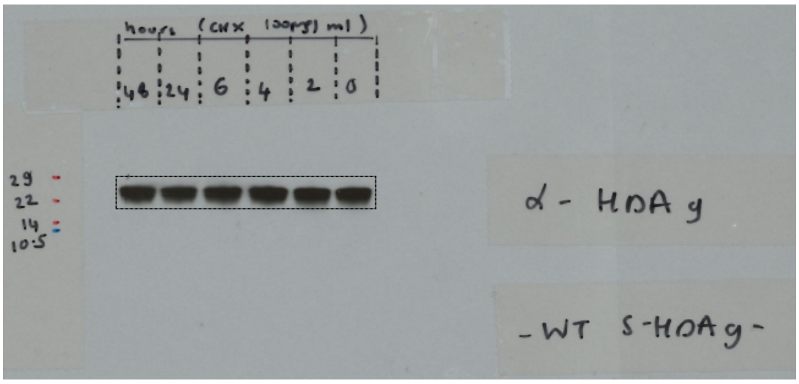

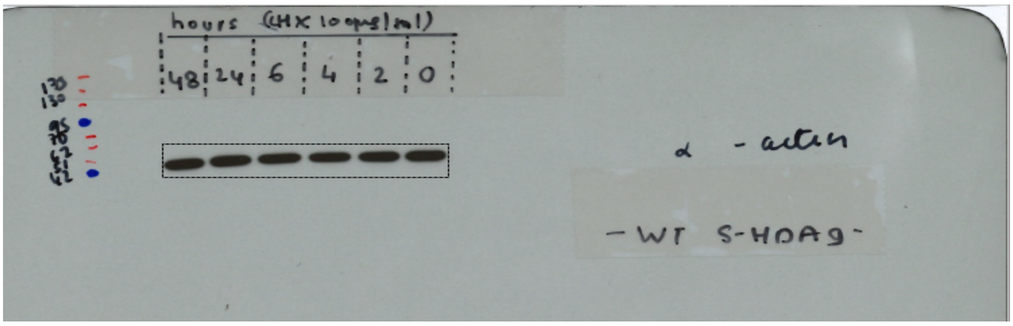

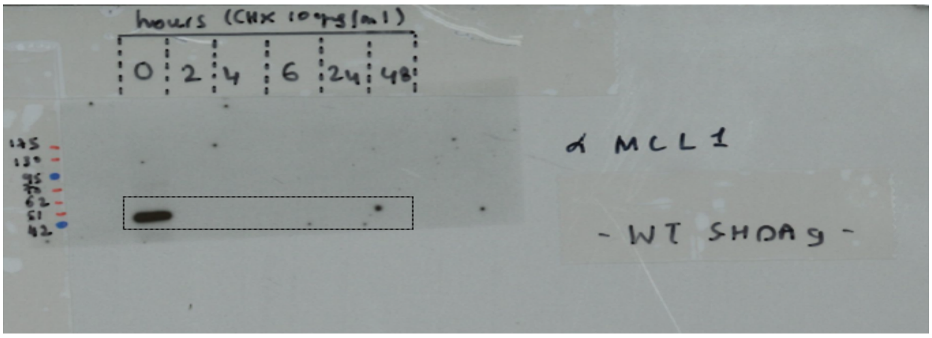

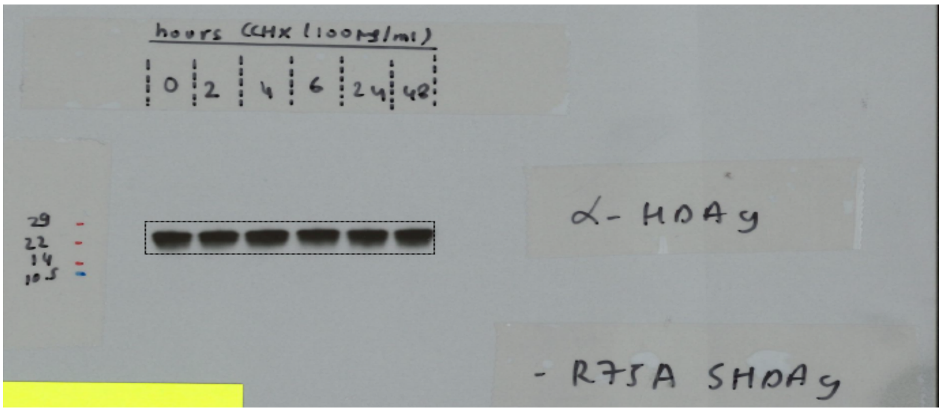

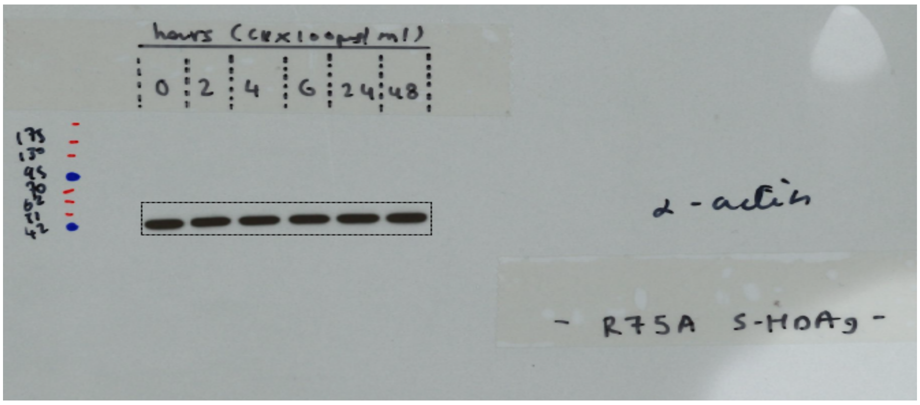
hours (CHX 100µg/ml)
0 2 4 6 24 48
α-actin
- R75A S-HDAg -

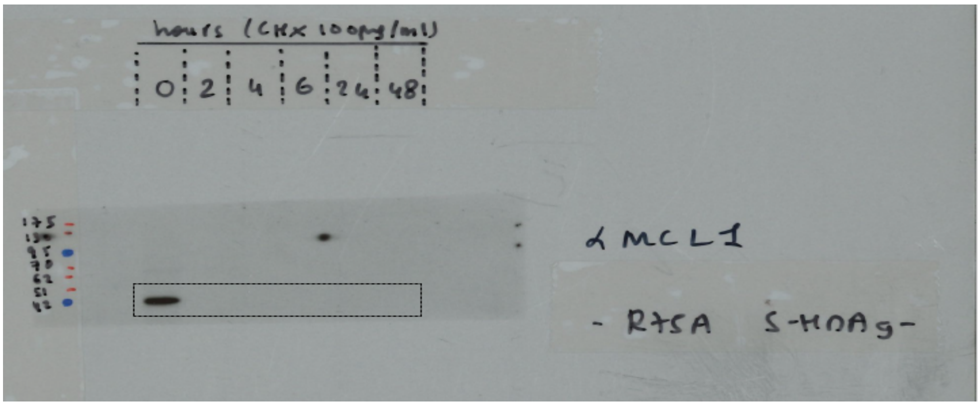
hours (CHX 100µg/ml)
0 2 4 6 24 48
α MCL1
- R75A S-HDAg -

Figure 3. R75A S-HDAg mutation affects the binding to BAZ2B BRD- without altering S-HDAg localization and acetylation.

Fig. 3f Co-immunoprecipitation of SHDAg and GFP-Tag-BAZ2B BRD

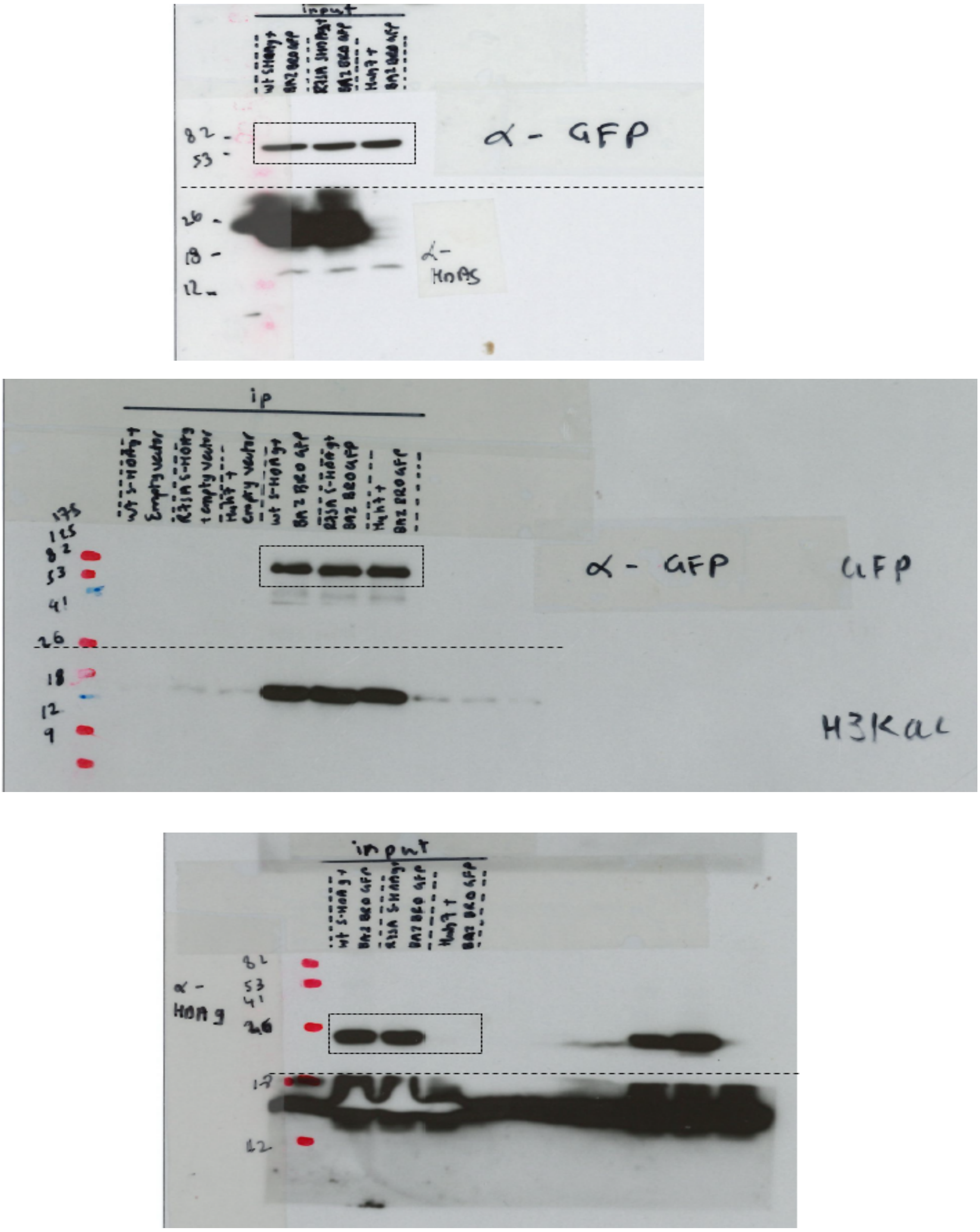

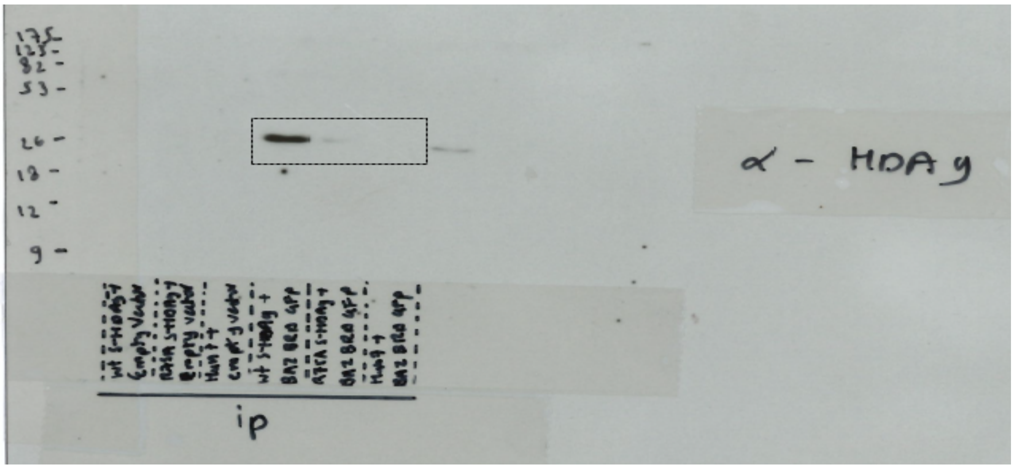

175
125
82
53
26
18
12
9
ip
α - HDAg

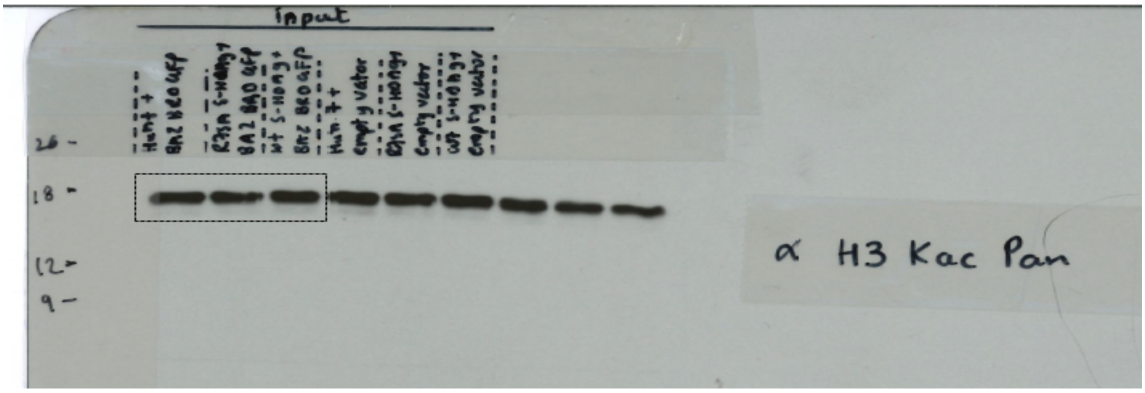

Input
26
18
12
9
α H3 Kac Pan

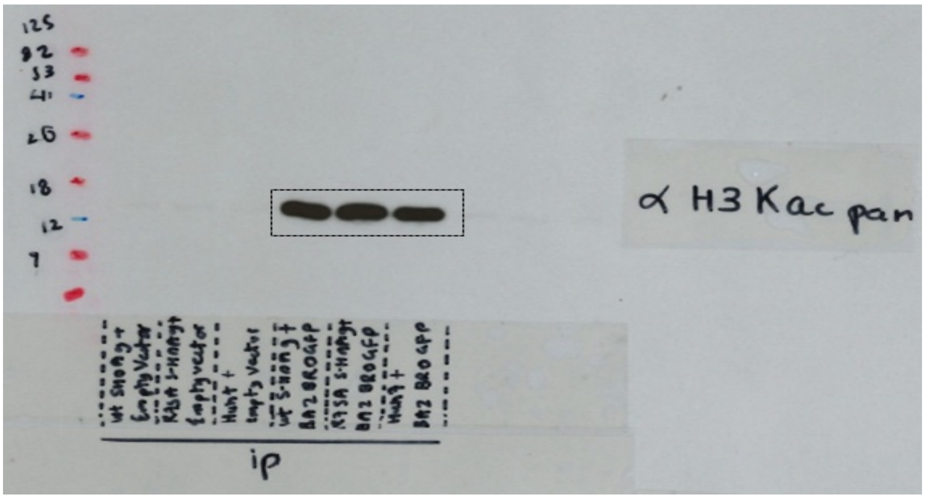

125
82
53
41
26
18
12
7
ip
α H3 Kac pan

Figure 4. Loss of HDAg Binding to BAZ2B Impairs HDV Replication

Fig. 4a Representative northern blot indicating the decrease in the accumulation in the presence of the R75A S-HDAg protein g HDV RNA

Northern blot assay (top panel)

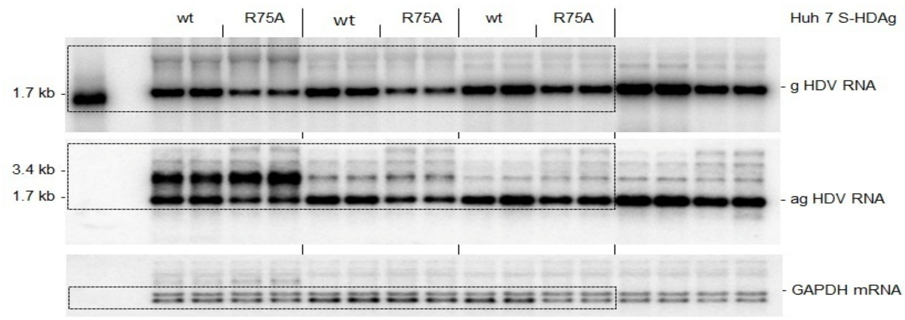

Northern blot histograms values (lower panel)

Genomic

	Huh 7 S-HDAg WT % g HDV RNA				
	Dataset 1	Dataset 2	Dataset 3	Mean	SEM
Day 3	100	100	100	100	0
Day 6	100	100	100	100	0
Day 9	100	100	100	100	0

	Huh 7 S-HDAg R75A % g HDV RNA				
	Dataset 1	Dataset 2	Dataset 3	Mean	SEM
Day 3	34	48	21	34,3333333	7,79601038
Day 6	31	48	66	48,3333333	10,1050043
Day 9	63	73	44	60	8,50490055

Multiple comparison test using Holm-Sidak correction method

	Significant?	P value	Mean1	Mean2	Difference	SE of difference	t ratio	df	Adjusted P Value
Day 3	Yes	0,00016552	100	34,33	65,67	6,523	10,07	5	0,00049646
Day 6	Yes	0,00170025	100	48,33	51,67	8,454	6,111	5	0,00339762
Day 9	Yes	0,00246644	100	60	40	7,116	5,621	5	0,00339762

Antigenomic

	Huh 7 S-HDAg WT % ag HDV RNA				
	Dataset 1	Dataset 2	Dataset 3	Mean	SEM
Day 3	100	100	100	100	0
Day 6	100	100	100	100	0
Day 9	100	100	100	100	0

	Huh 7 S-HDAg R75A % ag HDV RNA				
	Dataset 1	Dataset 2	Dataset 3	Mean	SEM
Day 3	49	39	59	49	5,77350269
Day 6	69	59	79	69	5,77350269
Day 9	79	73	85	79	3,46410162

Multiple comparison test using Holm-Sidak correction method

	Significant?	P value	Mean1	Mean2	Difference	SE of difference	t ratio	df	Adjusted P Value
Day 3	Yes	0,00090658	100	49	51	5,774	8,833	4	0,00271727
Day 6	Yes	0,00580981	100	69	31	5,774	5,369	4	0,00746292
Day 9	Yes	0,00373845	100	79	21	3,464	6,062	4	0,00746292

Northern blot dataset 3 (g HDV RNA)

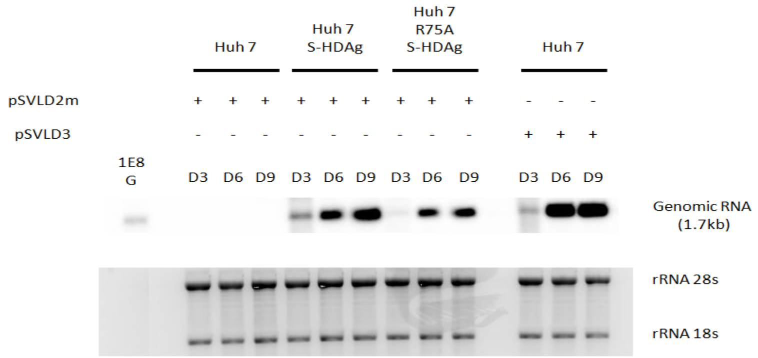

Northern blot dataset 3 (ag HDV RNA)

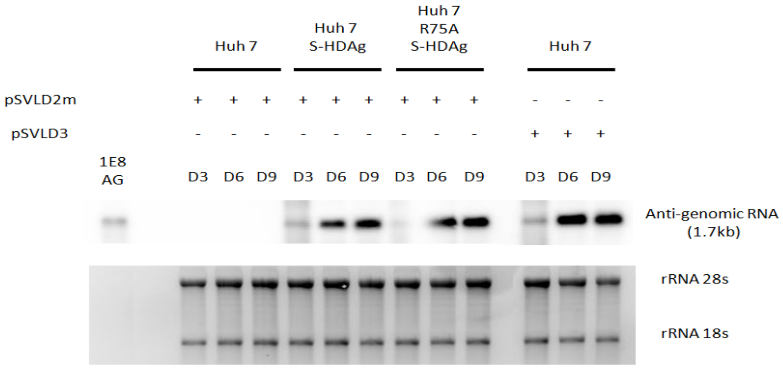

Figure 4. Loss of HDAg Binding to BAZ2B Impairs HDV Replication

Fig 4b. PHHs infection with wt HDV or recombinant R75A HDV mutant

Protein analysis (Western blot) - upper panel

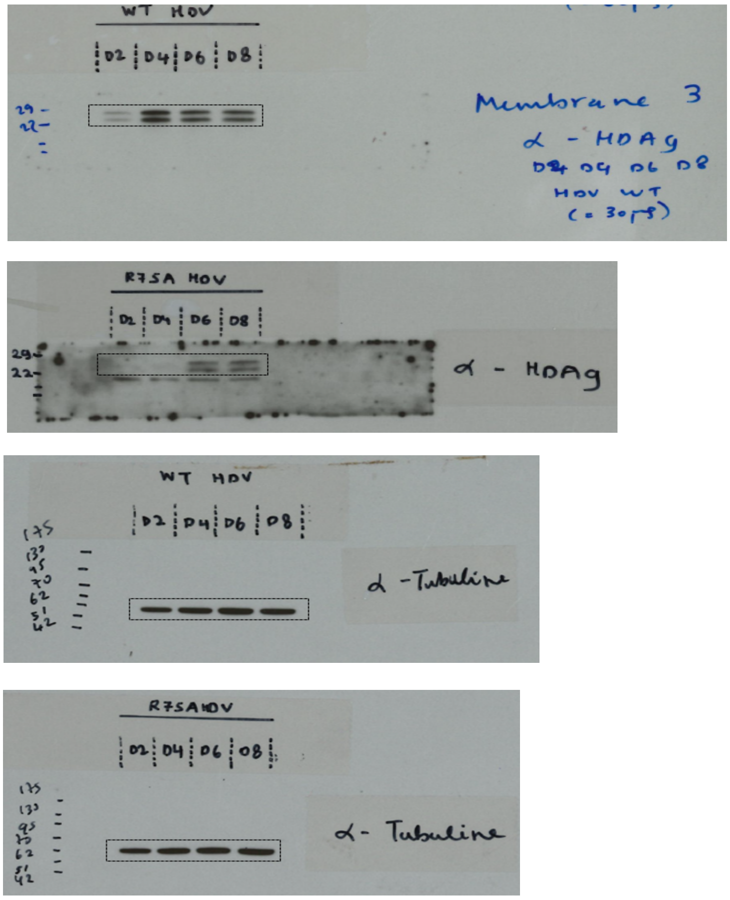

RNA analysis (Northern blot assay) - middle panel

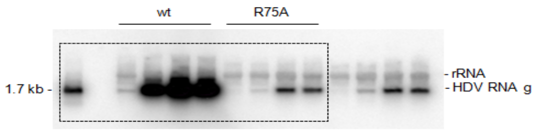

Northern blot histogram values (lower panel)

	Huh 7 S-HDAg WT % g HDV RNA				
	Dataset 1	Technical replicate dataset 1	Dataset 2	Mean	SEM
Day 2	7130518,8	7122443,32	7140618,75	7131193,606	5257,63457
Day 4	491275758	385755987	280382989	385804911,2	60879503,39
Day 6	1,173E+09	1018906621	868640477	1020274780	87943719,77
Day 8	973018989	887250503	806531908	888933800,1	48068049,51

	Huh 7 S-HDAg WT % g HDV RNA				
	Dataset 1	Technical replicate dataset 1	Dataset 2	Mean	SEM
Day 2	2592619,2	1868824,97	1157170,31	1872871,507	414383,3525
Day 4	2273770,7	2843927,37	3418777,43	2845491,826	330535,9033
Day 6	39027686	27860633,4	16696939,6	27861753,08	6446331,305
Day 8	32885383	26124969,1	19393171,6	26134507,87	3894868,833

Multiple comparison test using Holm-Sidak correction method

	Significant?	P value	Mean1	Mean2	Difference	SE of difference	t ratio	df	Adjusted P Value
Day 2	Yes	0,000222	7131194	1872872	5258322	414417	12,69	4	0,000666
Day 4	Yes	0,003263	385804911	2845492	382959419	60880401	6,29	4	0,003263
Day 6	Yes	0,000355	1020274780	27861753	992413027	88179663	11,25	4	0,00071
Day 8	Yes	0,000057	888933800	26134508	862799292	48225589	17,89	4	0,000229

Norther blot (dataset 2)

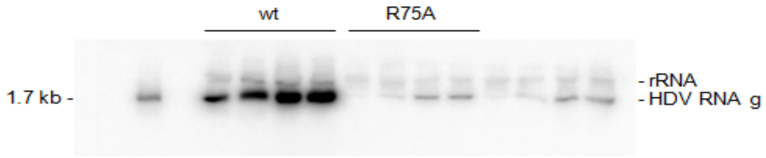

Figure 4. Loss of HDAg Binding to BAZ2B Impairs HDV Replication

Fig. 4c HDV RIP in wt HDV or R75A HDV infected PHHs

	wt HDV (% Input)	
	Dataset 1	Dataset 2
HDAg	80,14851	64,54987
Pol II	28,19531	22,97153
BAZ2B	5,273036	3,360893
SNF2L	8,824644	8,458326
H3	0,2	0,2

	R75A HDV (% Input)	
	Dataset 1	Dataset 2
HDAg	24,04677	14,56504
Pol II	2,822522	1,497588
BAZ2B	0	0
SNF2L	1,016885	1,00023
H3	0,2	0,2

Supplementary Figure 1. Characterization of the HepaRG cell line expressing StrepTag (ST) S-HDAg protein under the control of a tetracycline inducible promoter

Fig S1a. Dose-dependent induction of the ST_S-HDAg protein

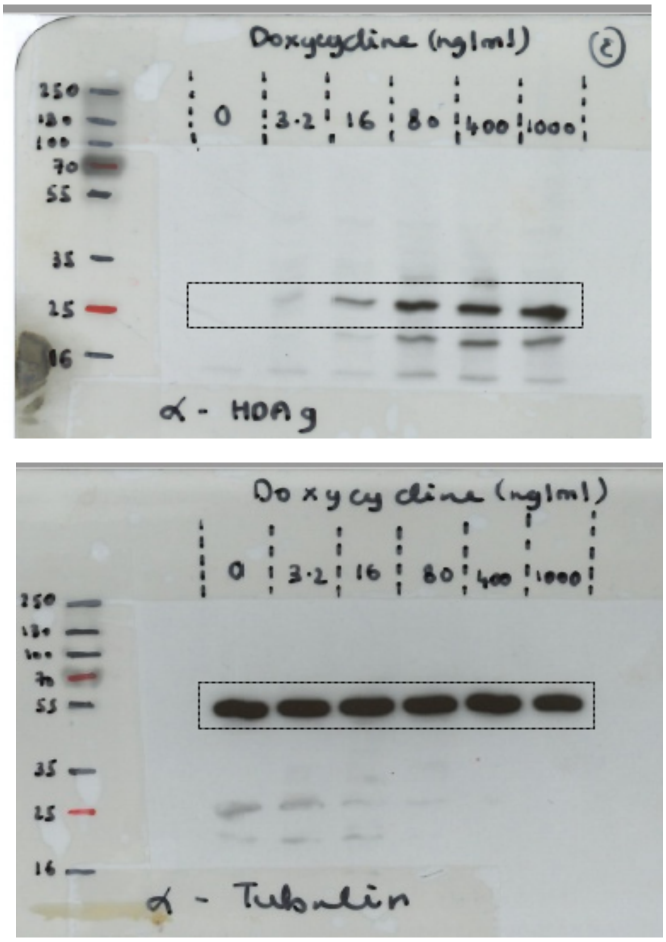

Supplementary Figure 1. Characterization of the HepaRG cell line expressing StrepTag (ST) S-HDAg protein under the control of a tetracycline inducible promoter

Fig S1b. Time-dependent induction of ST_S-HDAg protein expression

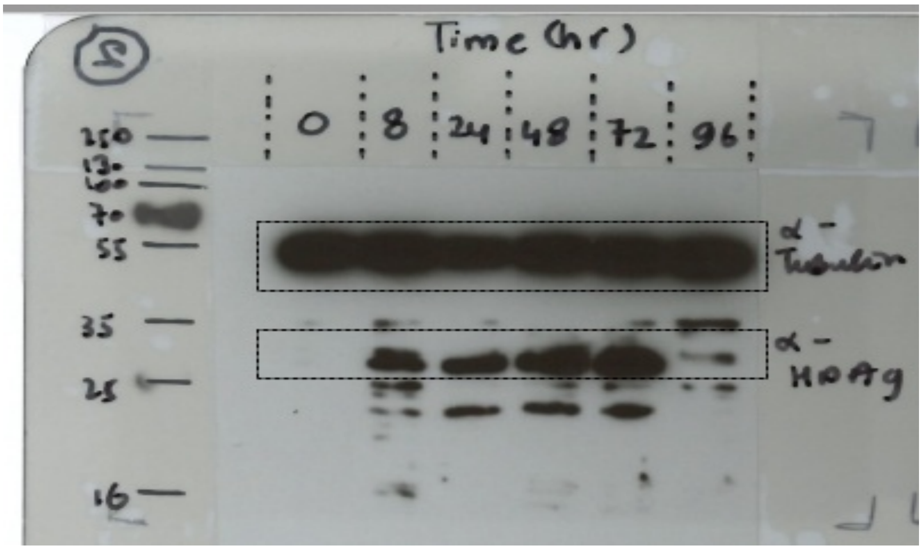

Supplementary Figure 1. Characterization of the HepaRG cell line expressing StrepTag (ST) S-HDAg protein under the control of a tetracycline inducible promoter

Fig. S1c The ST_S-HDAg protein is predominantly localized in the chromatin bound fraction

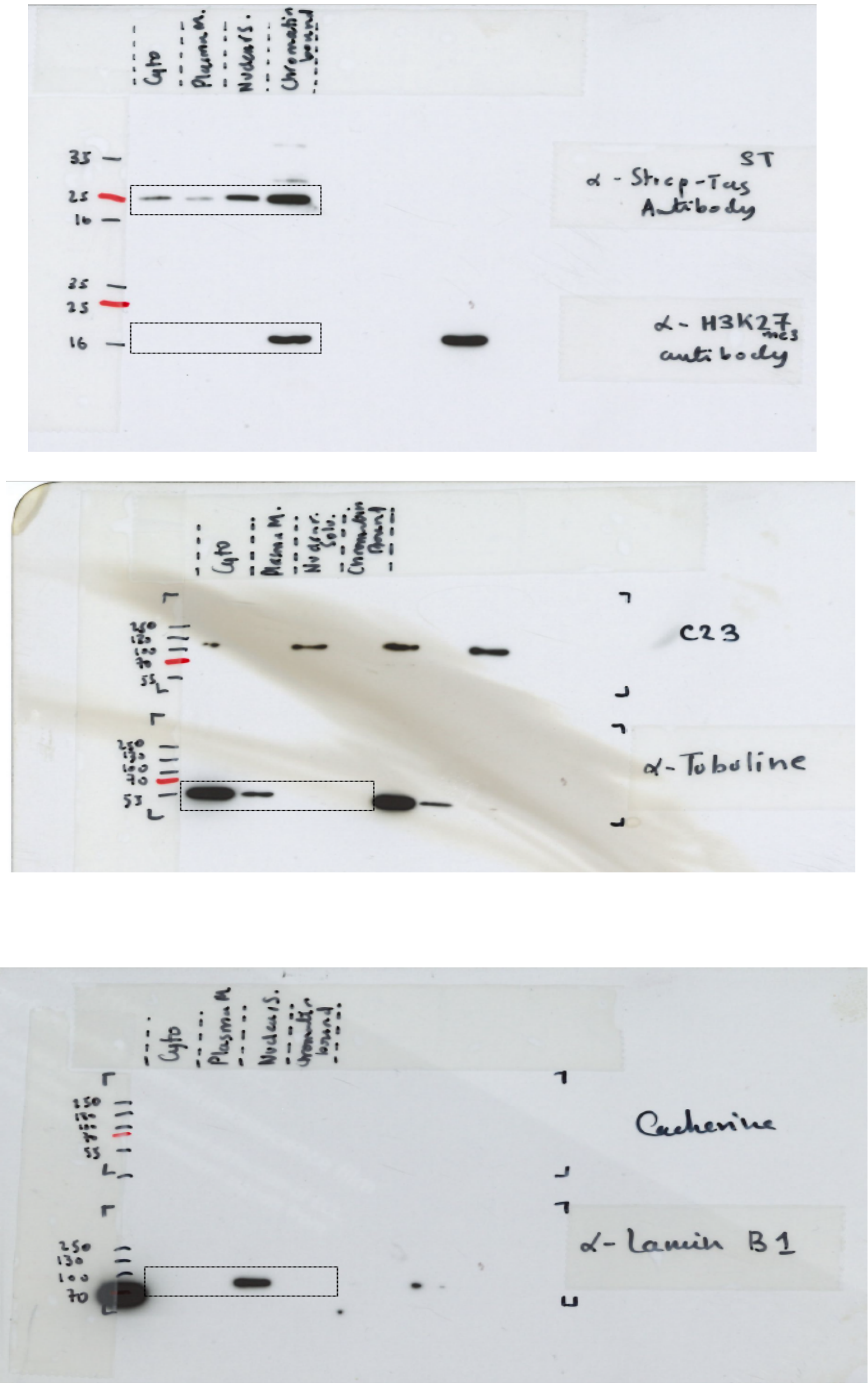

Supplementary Figure 1. Characterization of the HepaRG cell line expressing StrepTag (ST) S-HDAg protein under the control of a tetracycline inducible promoter

Fig. S1d The ST_S-HDAg protein accumulates in the nucleus

Provided upon request

(The files could not be enclosed since they were too heavy to be uploaded)

Supplementary Figure 1. Characterization of the HepaRG cell line

Fig. S1e The ST-S-HDAg protein is functional in HDV replication

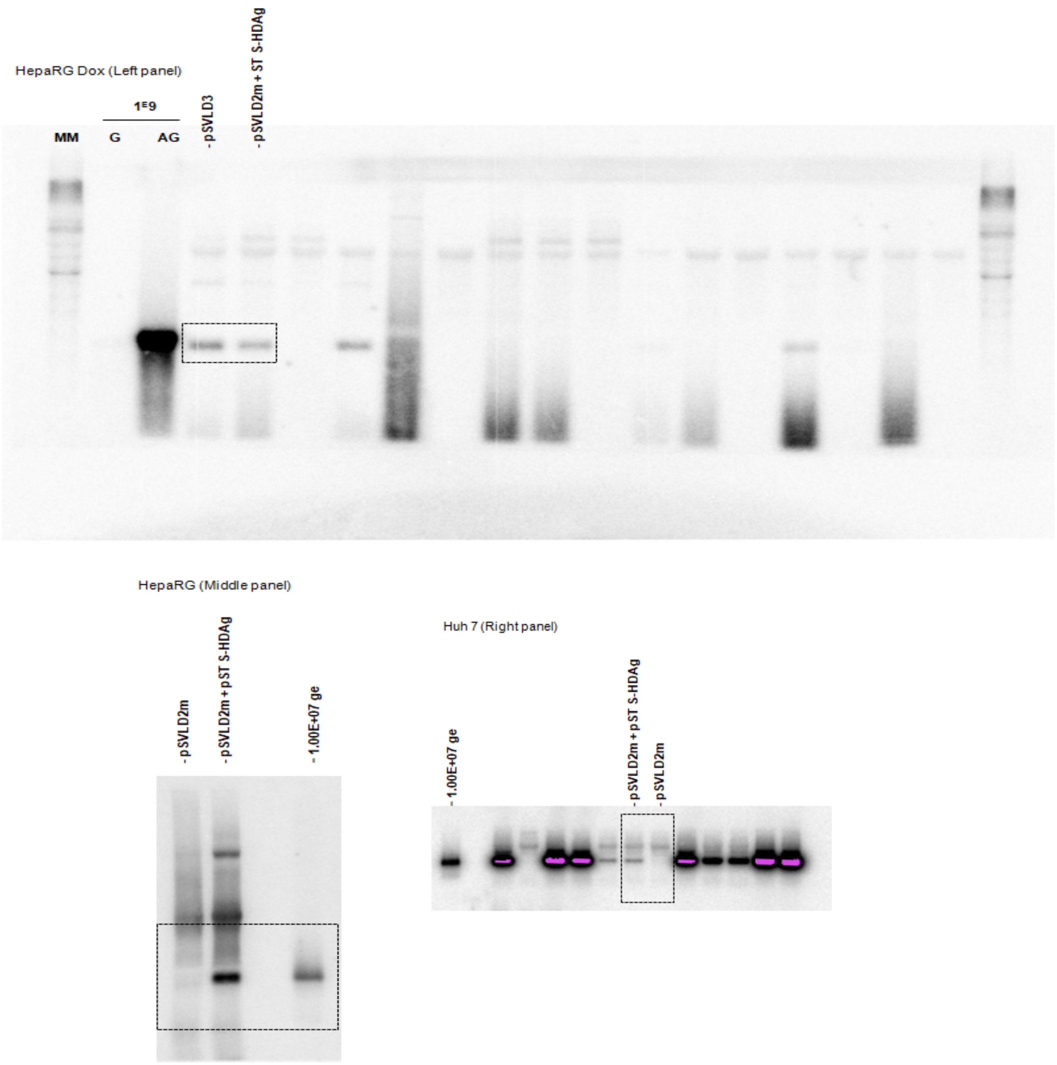

Supplementary Figure 4. ST-S-HDAg affinity purification on Strep-Tactin® coated beads

Fig S4a. The ST-S-HDAg was subjected to affinity chromatography on a Strep-Tactin®-XT resin

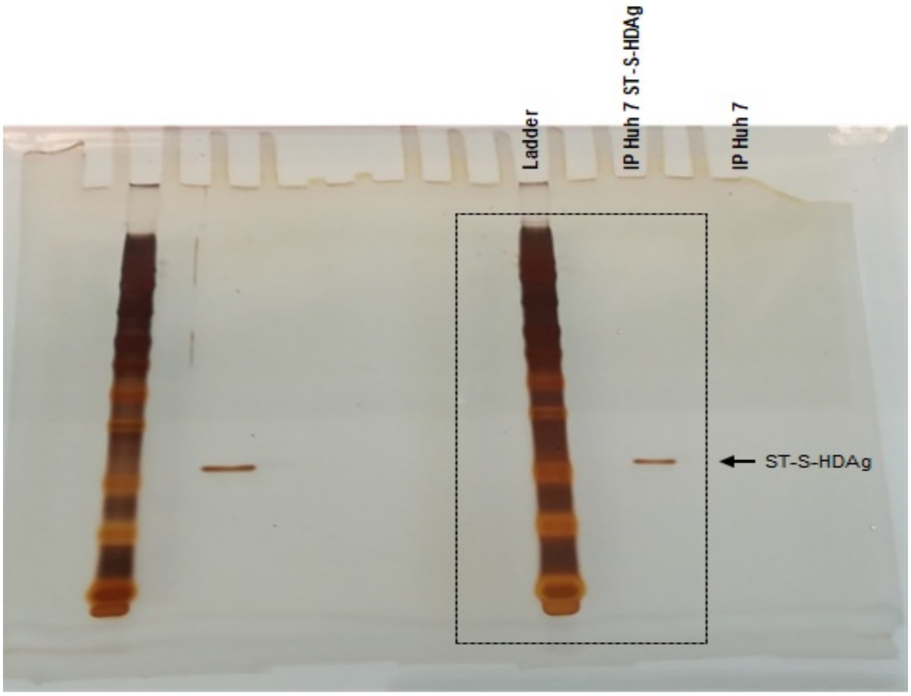

Supplementary Figure 4. ST-S-HDAg affinity purification on Strep-Tactin® coated beads

Fig S4b. ST-S-HDAg binds specifically to the StrepTactin®-XT beads

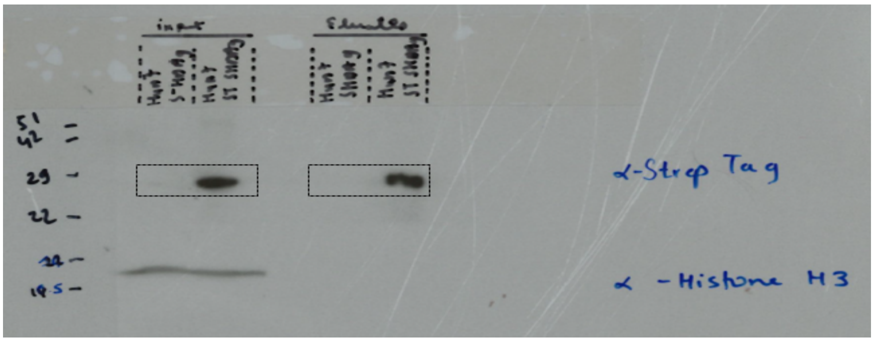

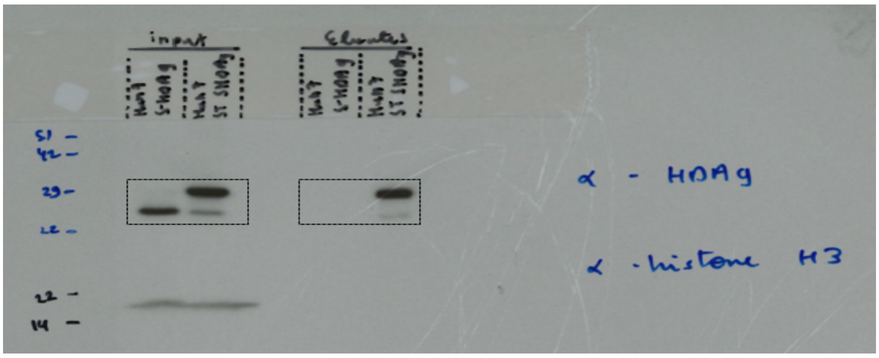

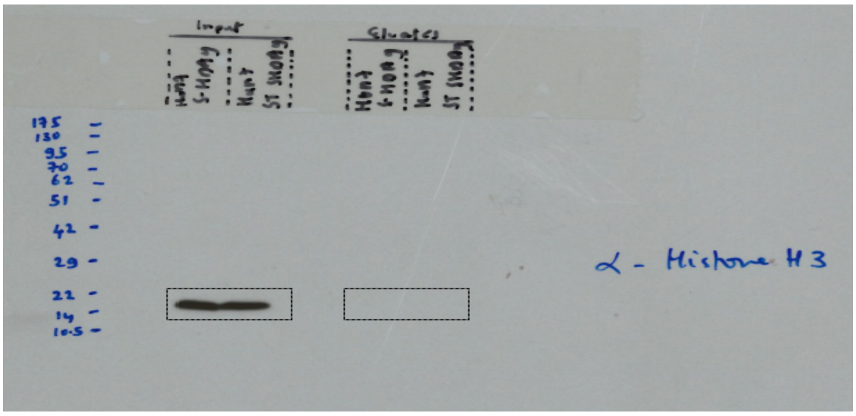

Supplementary Figure 4. ST-S-HDAg affinity purification on Strep-Tactin® coated beads

Fig S4c. Pull-down assay of His-Tag BAZ2B BRD and ST-S-HDAg

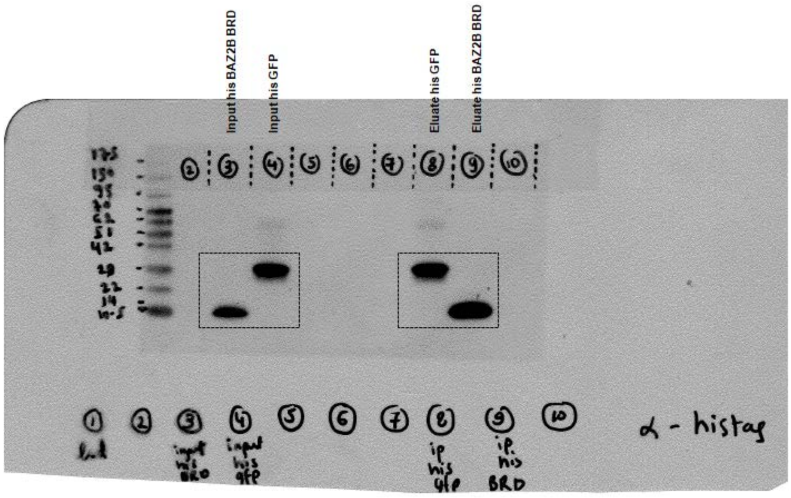

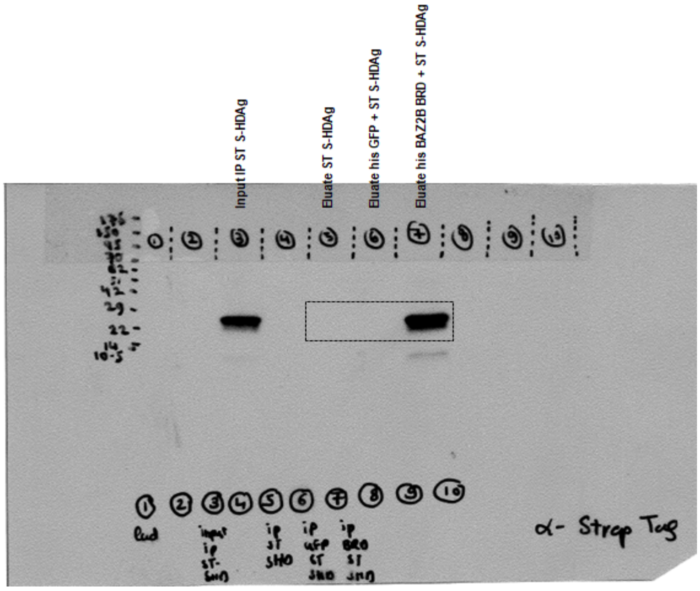

Supplementary Figure 5. Recombinant virus production, calibration and infection of the Huh7-106 cell line.

Fig S5a. Viral production

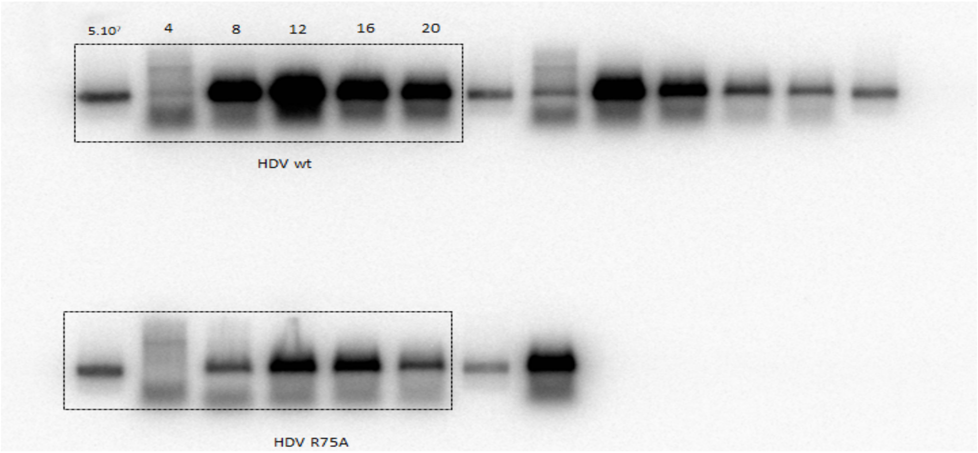

Supplementary Figure 5. Recombinant virus production, calibration and infection of the Huh7-106 cell line.

Fig S5b. Northern blot and and immunblot were performed on viral preparations to standardize the inoculum for both viruses

HDV RNA

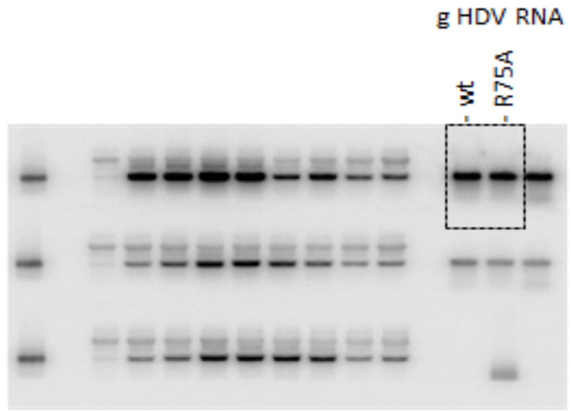

HDAg proteins

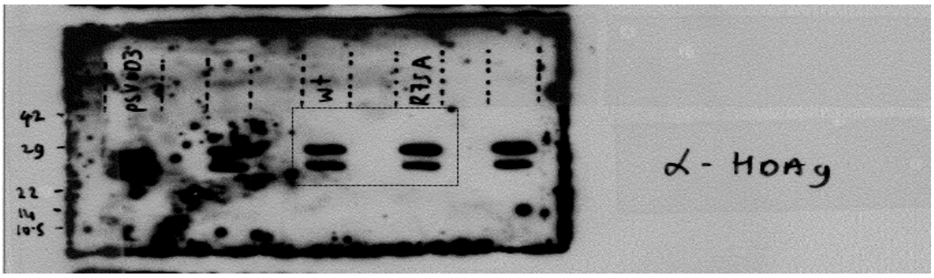

Supplementary Figure 5. Recombinant virus production, calibration and infection of the Huh7-106 cell line.

Fig S5c. Viral infection of Huh7-106 cell line

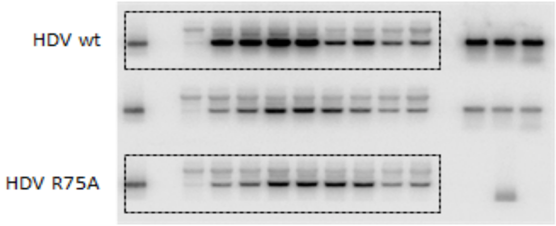

Supplementary Figure 5. Recombinant virus production, calibration and infection of the Huh7-106 cell line.

Fig S5d. Antigenomic (ag) HDV RNA was detected using a genomic riboprobe in total cell RNA prepared

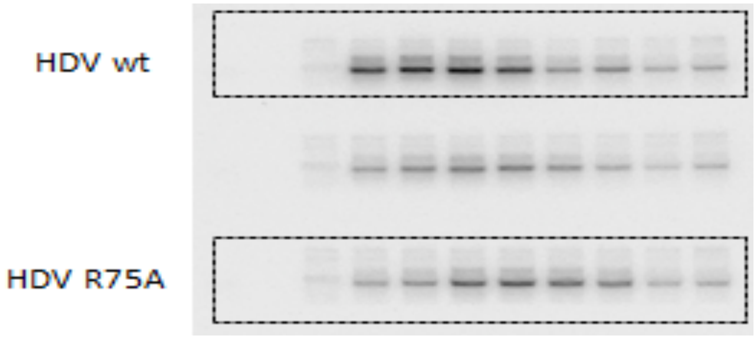

Supplement: Supplementary file 3 — Source Data [file 41467_2020_14299_MOESM3_ESM.pdf]
